# ABCtoolbox: a versatile toolkit for approximate Bayesian computations

**DOI:** 10.1186/1471-2105-11-116

**Published:** 2010-03-04

**Authors:** Daniel Wegmann, Christoph Leuenberger, Samuel Neuenschwander, Laurent Excoffier

**Affiliations:** 1 Institute of Ecology and Evolution, University of Bern, 3012 Bern, Switzerland; 2 Swiss Institute of Bioinformatics, 1015 Lausanne, Switzerland; 3 Ecole d'ingénieurs de Fribourg, 1705 Fribourg, Switzerland; 4 Department of Ecology and Evolution, University of Lausanne, CH-1015 Lausanne, Switzerland

## Abstract

**Background:**

The estimation of demographic parameters from genetic data often requires the computation of likelihoods. However, the likelihood function is computationally intractable for many realistic evolutionary models, and the use of Bayesian inference has therefore been limited to very simple models. The situation changed recently with the advent of Approximate Bayesian Computation (ABC) algorithms allowing one to obtain parameter posterior distributions based on simulations not requiring likelihood computations.

**Results:**

Here we present ABCtoolbox, a series of open source programs to perform Approximate Bayesian Computations (ABC). It implements various ABC algorithms including rejection sampling, MCMC without likelihood, a Particle-based sampler and ABC-GLM. ABCtoolbox is bundled with, but not limited to, a program that allows parameter inference in a population genetics context and the simultaneous use of different types of markers with different ploidy levels. In addition, ABCtoolbox can also interact with most simulation and summary statistics computation programs. The usability of the ABCtoolbox is demonstrated by inferring the evolutionary history of two evolutionary lineages of *Microtus arvalis*. Using nuclear microsatellites and mitochondrial sequence data in the same estimation procedure enabled us to infer sex-specific population sizes and migration rates and to find that males show smaller population sizes but much higher levels of migration than females.

**Conclusion:**

ABCtoolbox allows a user to perform all the necessary steps of a full ABC analysis, from parameter sampling from prior distributions, data simulations, computation of summary statistics, estimation of posterior distributions, model choice, validation of the estimation procedure, and visualization of the results.

## Background

Bayesian statistics has gained popularity in scientific inference, especially in population genetics and genomics [1]. Consider a model creating data D determined by parameters **θ **whose joint prior density is denoted by π (**θ**). The quantity of interest is the posterior distribution of the parameters **θ**, which is given by Bayes theorem as π (**θ**|D)~f(D|**θ**)π (**θ**), where f(D|**θ**) is the likelihood of the data. Unfortunately the evaluation of likelihoods is often difficult or even impossible for complex models. However, Monte-Carlo simulations can be used to approximate the likelihood function. For instance, a simple rejection algorithm has been proposed [2-4] to estimate the likelihood: a candidate parameter vector **θ **is simulated from a prior distribution and accepted if the corresponding vector of summary statistics **S **is sufficiently "close" to the observed summary statistics **S**_obs _with respect to some metric in the space of S (i.e., if ||**S **- **S**_obs_|| ≤ ε for a fixed tolerance ε). The precision of the posterior estimate will improve with smaller values of ε, but small ε values are often associated with very small acceptance rates (that are proportional to the likelihood) and will thus require many more computations.

More recently, Beaumont *et al*. [5] introduced a simple post-sampling adjustment to improve the estimation of posteriors from the joint distribution of accepted parameter and summary statistics vectors. They introduced a locally-weighted linear regression of parameters on summary statistics around the observed summary statistics, which allows one to obtain good estimations with relatively large acceptance intervals. Additional improvements have been proposed to more efficiently explore the parameter space, including several variations of likelihood-free MCMC [6-9] or Particle-based samplers [10-12]. All these methods are generally referred to as Approximate Bayesian Computations (ABC) and reviewed in [13,14].

Here, we present ABCtoolbox, a series of computer programs that can be pipelined to estimate parameters of complex models. In complement to available ABC packages [15-17], ABCtoolbox incorporates several ABC algorithms, handles various types of data by interacting with external simulation programs and includes tools to rigorously validate estimations, a necessary step of any Bayesian inference. ABCtoolbox comes with a detailed manual introducing all implemented algorithms and giving hints on how to successfully apply ABC inference. We demonstrate the usability of ABCtoolbox by inferring sex-specific migration rates and population sizes of the common vole *Microtus arvalis*.

## Implementation

**Figure 1:**
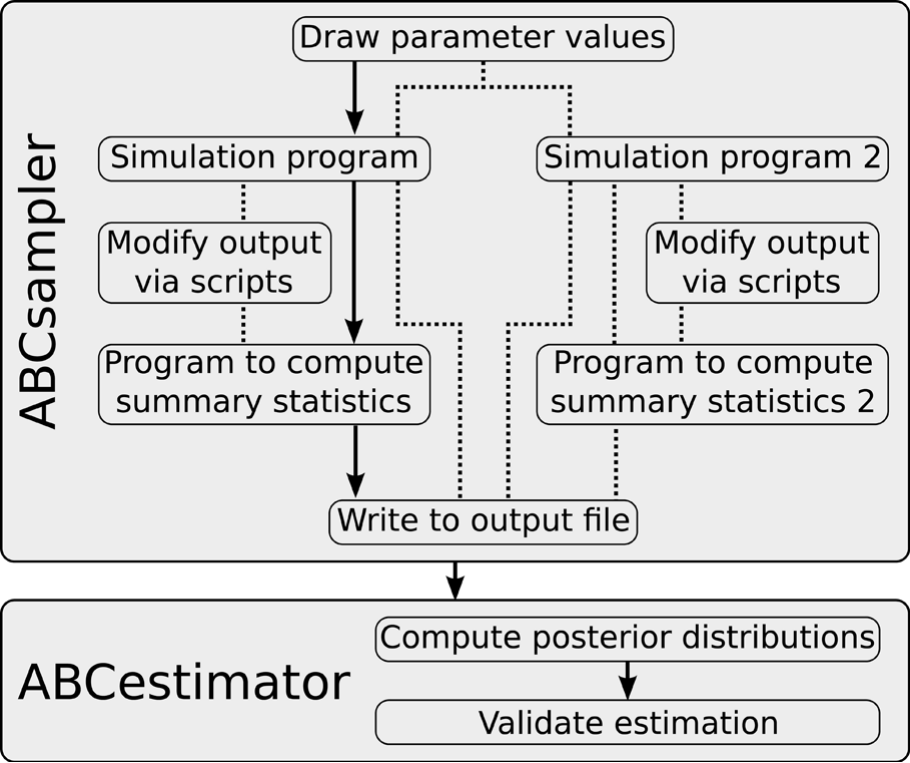
**Flowchart describing the individual steps of an ABC estimation by *ABCtoolbox***. Black arrows indicate the standard approach. Some alternative paths are shown with dotted lines. For instance, it is possible to modify the output of a simulation program such as to allow one to take specific characteristics of the observed data into account such as a given level of missing data. Additionally, *ABCtoolbox *can call several simulation programs per iteration, each of which can be launched with the same parameter values. Thus, different data types can be conveniently combined in a single analysis.

An ABC estimation is typically done in two steps: a large number of simulations are first carried out (simulation step) and then used to estimate posterior distributions (estimation step). The package incorporates two main programs for these two steps (Figure 1): *ABCsampler *generates the simulations and computes summary statistics via ancillary programs, and *ABCestimator *calculates marginal posterior distributions of parameters from recorded simulations, with or without regression adjustment. Both programs are implemented in C++ and can be fully parameterized via input files or the command line, which makes our package suitable for being used on computer grids or clusters. We will now outline the functionality of both programs in more detail.

*ABCsampler *(i) samples model parameter values (ii) calls an external simulation program, (iii) calls an external program computing summary statistics on simulated data, and (iv) writes parameter values and resulting summary statistics to a file. These four basic steps are then iterated many times until a large set of simulations is generated. The main parameters for *ABCsampler *are the names of the simulation program and the program to compute summary statistics, and the ABC sampler to be used. The sampling of parameter values can either be done using a rejection algorithm [3], a likelihood free MCMC [9] or a Population Monte Carlo sampler [11]. We refer the reader to the original papers and to the *ABCtoolbox *user manual for details on the implemented algorithms. Due to the fully object orientated implementation of *ABCsampler*, it would be straightforward to include additional sampling algorithms such as likelihood free MCMC with state-space augmentation [7] or to adopt a tempering scheme for the likelihood free MCMC [6].

Interaction of *ABCsampler *with external programs is done via the command line or via input files, which allows one to use most of the many genetic data simulation programs available, such as SIMCOAL2 [18], SPLATCHE [19], ms [20] or FREGENE [21]. *ABCsampler *is also fully flexible concerning the number of programs to be called per iteration, which allows one to generate different types of data for the same model parameters (Figure 1). For instance, DNA sequences and microsatellites data with different ploidy levels can be generated by calling SIMCOAL2 twice with different parameter files as input. *ABCsampler *also offers the possibility to call any script or program to modify the output of the simulation program (Figure 1). This feature allows, for instance, to simulate observed patterns of missing data by simply removing simulated genotypes from the output of SIMCOAL2. *ABCsampler *can also deal with linear combinations of summary statistics like Partial Least Squares (PLS), where one computes a few orthogonal components in the summary statistics space best explaining the parameter variability [9,22].

The program *ABCestimator *directly reads the output of *ABCsampler *and computes posterior distributions based on a fraction of the simulations closest to the observed data. The regression adjustment implemented in *ABCestimator *follows a recent formulation termed ABC-GLM [23] that differs from that proposed by Beaumont et al. [5], but it also leads to the marginal posterior distribution of each parameter. In addition, it computes the marginal density of a model, which naturally leads to the computation of Bayes factors and performing Bayesian model choice [23]. *ABCestimator *further offers two ways to validate the estimation procedure. First, one can test the ability of ABC to estimate parameters by analyzing a large number of simulated data sets with known parameter values drawn from the prior distributions (generated using *ABCsampler*) [24]. Based on such a test dataset, *ABCestimator *computes accuracy measures, such as bias, mean squared errors and coverage properties [9]. Secondly, *ABCestimator *offers a new way to check if the observed data are in strong disagreement with the assumed model. The idea is to compute the distribution of the marginal densities for all simulations retained for posterior estimation. The marginal density of the observed data is then compared against this distribution to compute a p-value indicative of the ability of the model to reproduce the data.

*ABCtoolbox *is also bundled with several additional command line utilities: the command-line version of the program ARLEQUIN [25], *arlsumstat*, to compute summary statistics, an additional program to compute simple summary statistics on microsatellite data (*strStats*), a program to linear-transform summary statistics (*transformer*), a program to simulate data from general linear models (GLM) and R scripts to estimate PLS components and to visualize results. The functionalities and usage of *ABCtoolbox *are fully described in a user manual, which also includes a complete methodological references of all algorithms implemented in *ABCtoolbox *and many hints and suggestions on how to perform successful ABC parameter estimations.

## Results and Discussion

We demonstrate the use of *ABCtoolbox *by studying the history of the common vole *Microtus arvalis*. This small rodent is probably the most abundant European mammal [26] and it has become a model species to study recolonization processes in Europe after the last ice age [26-28]. The common vole has generally very limited dispersal abilities [29], and differences in migration patterns between males and females have been reported [28]. Here we use 11 available population samples from the Central (8 samples) and Eastern (3 samples) *M. arvalis *evolutionary lineages to infer sex-specific migration rates and population sizes, along with key parameters of the demographic history of these lineages. All 218 sampled individuals (between 16 and 25 per population) have been typed for 11 nuclear microsatellites and for 320 bp of the female transmitted mitochondrial DNA (mtDNA) control region [26,30].

### Assumed Model of *Microtus arvalis *evolution

**Figure 2:**
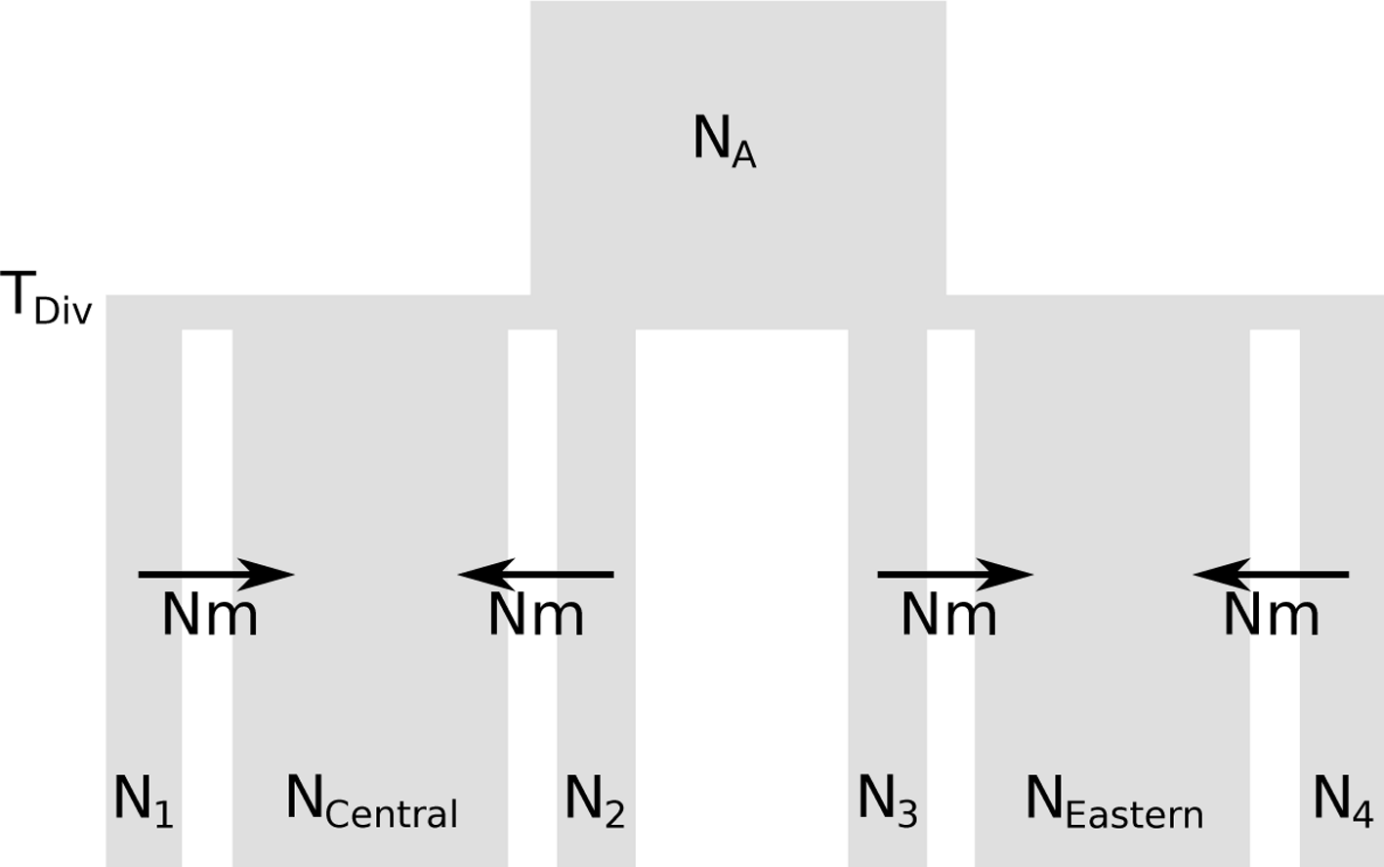
**Evolutionary model of the demographic history of two groups of populations corresponding to the Central and Eastern mtDNA evolutionary lineages of the common vole *Microtus arvalis***. An ancestral population of size *N*_*A *_diverges into the two population groups *T*_*DIV *_generations ago. We assumed a continent-island model for each of the two population groups. Islands (numbered subscripts) represent populations from which genetic data is available and the continents (subscripts "Eastern" and "Central") represent collectively all unsampled populations from a given evolutionary lineage. We further assumed the population sizes of the continents (*N*_Central _and *N*_Eastern_) to be large (10^7 ^individuals) and the population sizes of the islands (*N_1_*, *N_2_*, *N_3_*, etc.) to follow a Normal distribution with mean *N *and standard deviation *σ*_*N*_. Note that only four out of the 11 islands are shown. Backward in time, migration was only allowed from islands to the continent at rates *Nm*. While the same demographic model was assumed for both marker types, population sizes and migration rates were scaled differently (see text).

Following previous findings [26,28], we assumed an evolutionary model (Figure 2), where the Central and Eastern mtDNA lineages of *M. arvalis *diverged from a panmictic population T_DIV _generations ago. We describe here the model first for mtDNA. Each lineage is modeled as a large panmictic continent-island model where samples are taken from small islands. The effective size of the *i*-th island *N*_i _is assumed to be normally distributed with mean *N *and standard deviation *σ*_N_. All remaining (and unsampled) populations are collectively represented by a large panmictic continent (of arbitrary size set to 10^7 ^individuals). As commonly assumed in continent-island models, migration is only allowed from the islands to the continent looking backward in time, and is constant over time and of rate *N*_*m *_for each island. Under this model, sampled populations do not directly exchange genes, which implies that the most recent common ancestor of any pair of genes drawn from different populations is not found in any of the sampled populations. This seems a very reasonable assumptions given the huge number of *M. arvalis *populations.

We used SIMCOAL2 to perform genetic simulations and *arlsumstat *to compute summary statistics. *ABCsampler *called SIMCOAL2 twice per iteration, once to simulate mtDNA and once to simulate microsatellite data. The same parameter values were used for both calls, but all population sizes were scaled by 2(1+ *β*) and all migration rates were calculated as 2(*Nm*+ *Nm*_*males*_) for microsatellites, where *Nm*_*males *_is independently drawn from prior distribution. This parameterization simply indicates that the number of mtDNA genes is equal to the number of diploid females in the population and that mtDNA gene flow only occurs through females. Microsatellite diversity depends here on both male and female individuals, and we assumed that the number of autosomal genes is equal to two times the number of males and females in the population, the number of males potentially differing from that of females by a factor *β*. The prior distribution of mtDNA mutation rate *μ*_DNA _was based on previous estimates [26]. Locus-specific mutation rates for the microsatellites were assumed to be distributed as a Gamma (α, α/*μ*_STR_) [9,24], with α being considered as a nuisance parameter. All prior distributions are summarized in Table 1. Note that parameters with prior distributions defined on a logarithmic scale were estimated on the same scale. Note finally that we used an average of 2.5 generations per year [26,28] to translate divergence times into years.

**Table 1:** Characteristics of the prior and obtained posterior distributions.

Parameter	Prior	Mode	HPDI50^c^	HPDI90^c^
*N*	U [10, 500]	73.89	[32.12, 125.49]	[10.00, 238.53]
σ_*N*_	U [10, 200]	166.58	[126.48, 188.54]	[59.65, 200.00]
*N*_*A*_^*a*^	10^U [3,6.5]^	86,000	[46100, 153400]	[17800, 300000]
*β*^*a*^	10^U [-1,1]^	0.25	[0.16, 0.80]	[0.10, 2.80]
*Nm*^*a*^	10^U [-1.5,1]^	0.16	[0.10, 0.23]	[0.05, 0.35]
*Nm*_males_^*a*^	10^*U *[-1.5,1]^	1.11	[0.56, 1.91]	[0.26, 3.92]
*T*_*DIV*_^*b*^	U [40,000, 80,000]	18,600	[16600, 21800]	[16000, 28100]
*μ*_*DNA *_× 10^8^	U [10^-8^, 5*10^-7^]	8.37	[6.29, 11.14]	[3.19, 14.78]
*μ*_*STR *_× 10^4^	U [10^-5^, 5*10^-4^]	1.33	[1.09, 1.61]	[0.52, 2.39]
*α*	U 8.00 12.00	9.05	[8.60, 10.33]	[8.14, 11.52]

^a^The posterior distributions of these parameters were estimated on a logarithmic scale. The reported posterior characteristics were then transformed onto the natural scale.

^b ^Whereas the prior of the divergence time *T*_*DIV *_is expressed in generations, its posterior is expressed here in years for convenience, assuming 2.5 generations per year [26,28]

^c ^The Highest Posterior Density Interval HPDI is chosen as the continuous interval of parameter values with highest posterior density.

### Estimation Procedure and Validation

**Figure 3:**
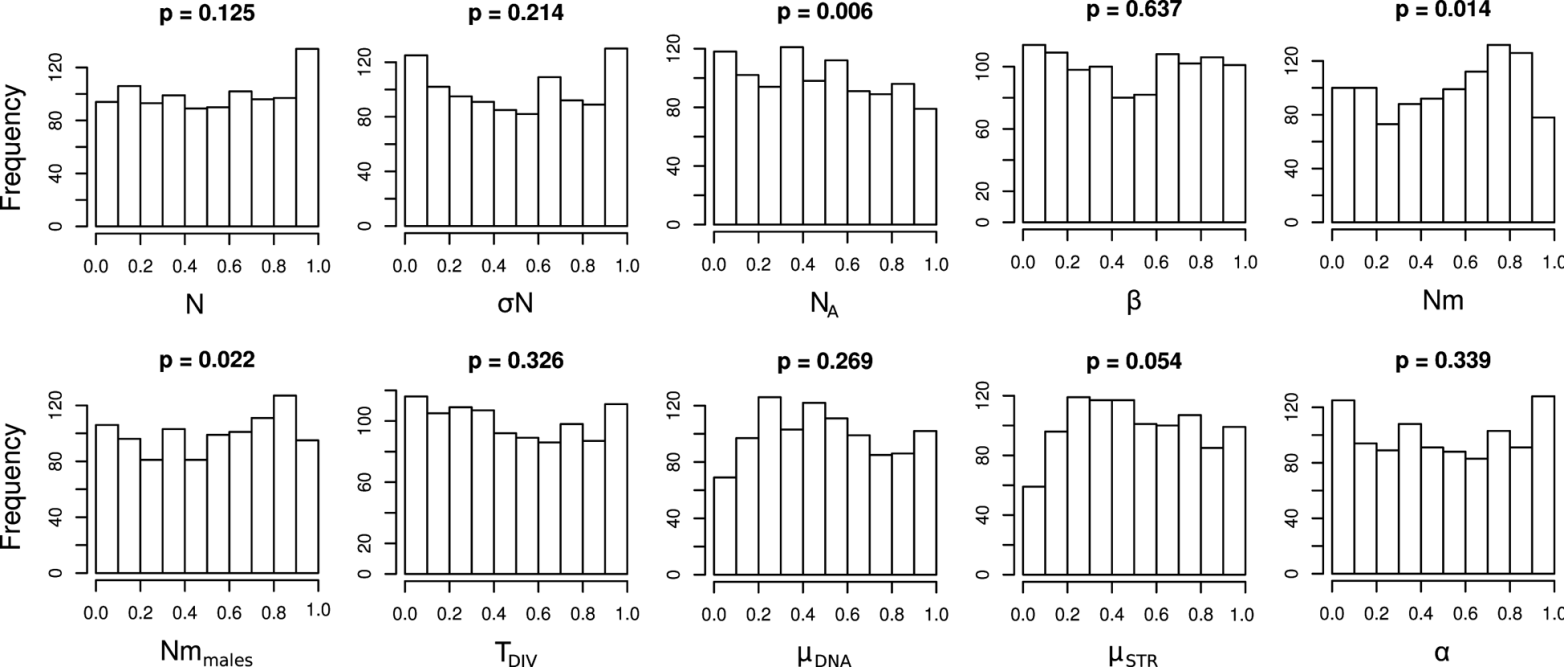
**Distributions of the quantiles (*x*-axis) of the known parameter values as inferred from the posterior distributions obtained with *ABCestimator *for 1000 pseudo-observed data sets**. These distributions are expected to be uniform if posterior densities have appropriate coverage properties [9]. We show these distributions for all model parameters (see text). The reported p-values above each histogram are the result of a Kolmogorov-Smirnov test for departure from distribution uniformity.

We computed a total of 338 summary statistics on the 11 populations and both mtDNA and microsatellite datasets, which were then reduced to 7 PLS components using specific R scripts of *ABCtoolbox*. Using *ABCsampler*, we ran a likelihood-free MCMC chain of 10^6 ^steps with tolerance *δ *= 0.1 and proposal range *ϕ *= 1, as defined in [9]. Posterior distributions were generated with *ABCestimator *under the ABC-GLM approach [23] using the best 5000 simulations. We validated the estimation procedure using *ABCestimator *based on 1000 datasets simulated with known parameter values generated with *ABCsampler*. In this validation step, posterior distributions were generated based on 10^6 ^simulations performed under a standard rejection sampling [2], as we could use the same set of 10^6 ^simulations to estimate parameters for all 1000 datasets. We report in Figure 3 the histograms of the posterior quantiles for each parameter, along with the *p*-value obtained by a Kolmogorov-Smirnov test of distribution uniformity [9]. We found none of these distributions to significantly differ from uniformity after correcting for multiple testing (Bonferroni correction), which suggests that our posteriors have an adequate coverage. In order to check if the observed data are in agreement with the simulated model, we computed the distribution of the marginal densities for the 5000 simulations retained for posterior estimation. The marginal density of the observed data was then compared against this distribution to compute a p-value (see above). We found the observed values to fall nicely within the simulated data (*p*-value 0.47), which suggests that the assumed model is capable of reproducing the observed summary statistics (the first 7 PLS components in our case).

### *Microtus arvalis *recent demographic history

**Figure 4:**
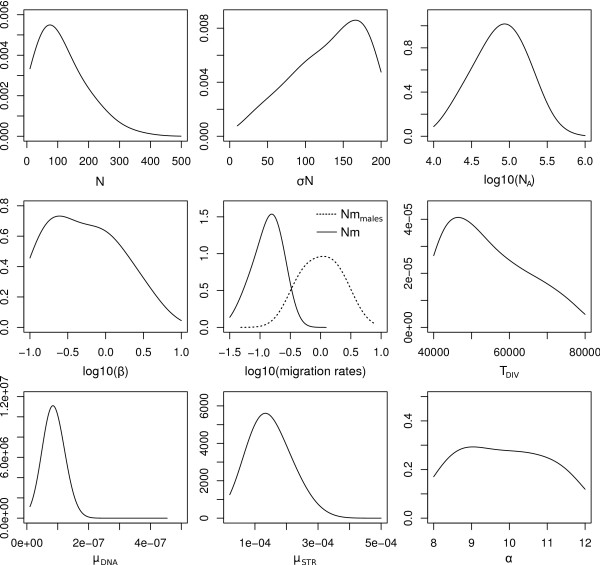
**Posterior distributions obtained with *ABCestimator *based on a likelihood-free MCMC chain of 10^6 ^steps performed by *ABCsampler***. Additional characteristics of the posterior distributions, along with the prior distributions, are given in Table 1. See Figure 2 and text for parameter description.

We report in Figure 4 posterior distributions of the parameters under the assumed evolutionary scenario described in Figure 2. Properties of these posteriors are summarized in Table 1. In keeping with previous results [28], we find that the number of male immigrants is ~7 times larger than that of females. Contrastingly female effective sizes are found four times larger than male effective sizes, which is in keeping with the large variance in reproductive success among *M. arvalis *males (J. Hahne, personal communication). Note however, that local population sizes are found to be extremely small on average (*N*_*f *_~75), but with a large standard deviation (~170), which is in good agreement with field observations [31]. Our results also support previous evidence [26] for a divergence between the central and the eastern lineage around the time of the last glacial maximum in Europe (T_DIV _~ 18,500 years). The strong demographic differences between males and females inferred here suggest that an incomplete picture may arise when studying colonization processes or inferring demographic history based on mtDNA alone.

## Conclusions

*ABCtoolbox *allows a user to perform all the necessary steps of a full ABC analysis, like parameter sampling from prior distributions, data simulations, computation of summary statistics, estimation of posterior distributions, model choice, validation of the estimation procedure, and visualization of the results. It includes various ABC algorithms, several of which are not found in any other available software package so far (e.g. likelihood-free MCMC [8,9], Population Monte Carlo [10-12] and ABC-GLM [23]), and the future addition of new algorithms is straightforward due to *ABCtoolbox*'s object-oriented design. In combination with SIMCOAL2 [18], *ABCtoolbox *is more flexible than available programs to perform ABC estimation of demographic parameters from genetic data [15,17] in that it allows (i) the simultaneous use of several data types (i.e. microsatellites, DNA sequences, or SNP data), each with potentially different ploidy levels and modes of transmission (ii) the inference of parameters under complex demographic scenarios including any combination of admixture, divergence and migration between an arbitrary number of populations with dynamic size changes, and (iii) the incorporation of additional features of the data, such as varying levels of missing data or population-specific inbreeding levels. However, *ABCtoolbox *is not tied to SIMCOAL2 and may equally well be used with other genetic simulation programs, like ms [20], SPLATCHE [19] or FREGENE [21]. It can thus allow parameter inference under spatially explicit models or models with natural selection. In fact, *ABCtoolbox *can interact with virtually any command-line simulation software and is capable of using several simulation programs per iteration, which could allow for instance the simultaneous analysis of different data sets or different types of data. The additional ability to use linear combinations of summary statistics best explaining parameter variability is also of interest when a prior selection of the most informative statistics is difficult.

The flexibility of *ABCtoolbox *has a cost in performance. Indeed, for each simulation, external programs may need to be launched, and communication between programs is mediated by text files, which is not optimal, and therefore simple models may be more quickly analyzed with canned ABC sampling software (e.g. [15,17]). However, this disadvantage may become negligible when considering the possibility of distributing simulations over the nodes of a cluster, or when simulation time of large data sets under complex models are much more costly than communication between subprograms. In any case, the overall speed of the computations primarily depends on the speed of the genetic simulations program. In this respect, we have used here SIMCOAL2 for its flexibility and ease of parameterization rather than for its speed, and the use of continuous-time coalescent simulations (such as ms [20]) should lead to much faster inferences.

## Availability and requirements

Project name: ABCtoolbox

Project home page: http://www.cmpg.iee.unibe.ch/

Operating system(s): Platform independent with supported Linux and Windows binaries

Other requirements: Windows users need to install the CYGWIN Linux-like environment for Windows, available on http://www.cygwin.com.

Programming language: C++ and R

License: GNU GPL version 3 or later

## Authors' contributions

DW, SN and LE designed and implemented ABCsampler. DW and CL designed and implemented ABCestimator. DW implemented all other programs and scripts. DW and LE performed the analysis and wrote the paper. All authors have read and approved the final manuscript.
